# The Functions of Non-coding RNAs in rRNA Regulation

**DOI:** 10.3389/fgene.2019.00290

**Published:** 2019-04-05

**Authors:** Qi Yan, Chengming Zhu, Shouhong Guang, Xuezhu Feng

**Affiliations:** ^1^Hefei National Laboratory for Physical Sciences at the Microscale, School of Life Sciences, University of Science and Technology of China, Hefei, China; ^2^CAS Center for Excellence in Molecular Cell Science, Chinese Academy of Sciences, Hefei, China

**Keywords:** rRNA, non-coding RNA, PAPAS, SLERT, 5S-OT, risiRNA, LoNA

## Abstract

Ribosomes are ribonucleoprotein machines that decode the genetic information embedded in mRNAs into polypeptides. Ribosome biogenesis is tightly coordinated and controlled from the transcription of pre-rRNAs to the assembly of ribosomes. Defects or disorders in rRNA production result in a number of human ribosomopathy diseases. During the processes of rRNA synthesis, non-coding RNAs, especially snoRNAs, play important roles in pre-rRNA transcription, processing, and maturation. Recent research has started to reveal that other long and short non-coding RNAs, including risiRNA, LoNA, and SLERT (among others), are also involved in pre-rRNA transcription and rRNA production. Here, we summarize the current understanding of the mechanisms of non-coding RNA-mediated rRNA generation and regulation and their biological roles.

## Introduction

In humans, there are approximately 300–400 copies of rDNA genes per haploid genome that are distributed over five chromosomes (Henderson et al., 1973; Boisvert et al., 2007). Each rDNA unit is ∼43 kb long. rDNAs are transcribed by RNA polymerase I (Pol I) to generate pre-rRNAs that subsequently undergo multiple modifications and processing steps to remove the external transcribed spacers (ETSs) and internal transcribed spacers (ITSs) to produce mature 18S, 5.8S, and 28S rRNAs (McStay, 2016). Pol I activity is a key determinant for ribosome abundance and is essential for cell growth and proliferation (McStay, 2006; Srivastava et al., 2016). Interestingly, only some rDNA units are transcriptionally active. Uncontrolled rRNA synthesis by dysregulated Pol I is associated with aberrant cell proliferation and oncogenesis (Peltonen et al., 2014; Nguyen le et al., 2015).

Non-coding RNAs play essential roles in rRNA regulation. The small nucleolar RNA (snoRNA) is widely known to guide the nucleotide modifications and processing (Cech and Steitz, 2014; Sloan et al., 2017). Recently, increasingly more studies have started to reveal the roles of other classes of non-coding RNAs in regulating rRNA transcription and nucleolar function (Srivastava et al., 2016; Bazin et al., 2017). In this review, we will focus on recent work investigating how several long non-coding RNAs (lncRNAs) and antisense ribosomal siRNA (risiRNA) regulate rRNA expression and their potential biological roles in anti-stress reactions (Table 1).

**Table 1:** Non-coding RNAs regulate rRNA production.

RNA	Classification	Synthesis	Organisms	Length	Function	References
snoRNA	snoRNA	Pol II	Eukaryotes	70 nt	Essential for pre-rRNA processing and modification by serving as a guide RNA	Cech and Steitz, 2014
risiRNA	siRNA	RdRP	*C. elegans*	22 nt	Suppresses pre-rRNA via the nuclear RNAi pathway to inhibit the accumulation of erroneous rRNAs	Zhou et al., 2017b; Zhu et al., 2018
pRNA	lncRNA	Pol I	Mouse, human	150∼300 nt	Complementary to rDNA promoters, required for the NoRC complex to suppress rRNA transcription	Bierhoff et al., 2010, 2014
LoNA	lncRNA	Pol II	Mouse	∼26 kb	Regulates rRNA transcription and methylation, involved in learning and memory	Li et al., 2018
SLERT	lncRNA	Pol II	Human	694 nt	Regulates DDX21 rings associated with Pol I transcription	Xing et al., 2017
PAPAS	lncRNA	Pol II	Mouse, human	17 kb	Interacts with chromatin remodelers at rDNA loci, responds to various environmental stresses	Bierhoff et al., 2014; Zhao et al., 2016a,b
5S-OT	lncRNA	Pol II	Fission yeast and mammals	847 nt	Regulates 5S rRNA production in cis and alternative splicing in trans	Hu et al., 2016


## lncRNAs Regulate rRNA Transcription in the Nucleus

Long non-coding RNAs comprise a fast-growing classes of RNA molecules with sizes greater than 200 nt. Most lncRNAs are first transcribed by polymerase II (Pol II), then capped, polyadenylated, and spliced after transcription (Cech and Steitz, 2014). lncRNAs localize in distinct subcellular compartments, including the nucleus, nucleolus and cytoplasm. Nuclear-localized lncRNAs, such as MANTIS and Xist, may function as transcriptional or posttranscriptional regulators or structural scaffolds for nuclear domains (Sun et al., 2017). The nucleolar localization of lncRNAs suggests that they may modulate rRNA transcription and maturation (Cech and Steitz, 2014; Chen, 2016). Several studies revealed that lncRNAs regulate rDNA transcription by altering rDNA epigenetic status or by acting as “decoys” to inhibit transcription factor activity. Interestingly, some lncRNAs might contain short open reading frames that can be translated (Ji et al., 2015).

### Binding of pRNAs to TIP5 Induces Heterochromatin Formation of rDNA Genes

**FIGURE 1:**
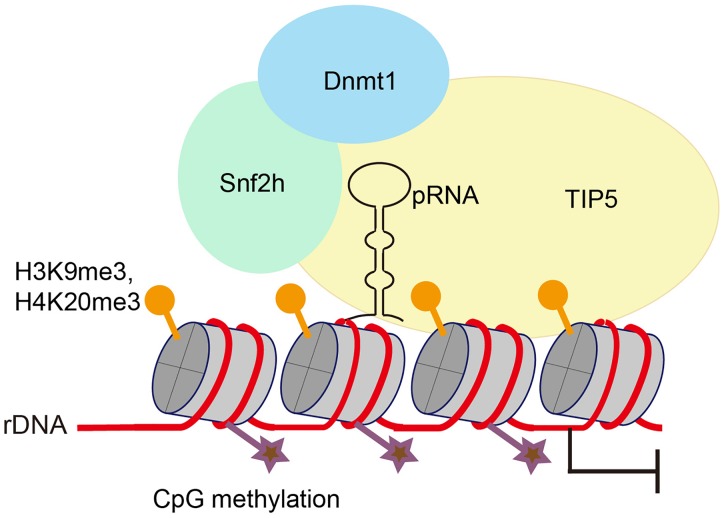
Promoter-associated RNA (pRNA) targets NoRC to nucleolus to promote heterochromatin formation and rDNA silencing. pRNA is complimentary to rRNA genes and folds into a stem-loop structure. TIP5, the core factor of NoRC, recognizes pRNA, facilitates formation of heterochromatin at rRNA genes and promotes transcriptional gene silencing.

There are several clusters of tandemly arrayed rDNA genes exist in each mammalian genome, yet not all of these repeats are transcribed. rDNA exists in two types of chromatin – a euchromatic conformation that is actively transcribed and a heterochromatic conformation that is transcriptionally inactive. Silent rDNA repeats are marked by heterochromatic histone modifications and CpG methylation at the rDNA promoter (Schmitz et al., 2010). Silencing of rDNA depends on NoRC, a chromatin-remodeling complex that directs heterochromatin formation. NoRC function requires RNA that is complementary to the rDNA promoter, which is termed as promoter-associated RNAs (pRNA). pRNAs are 150∼300 nt long and are produced from rDNA promoters. TIP5 (TIF interacting protein 5), the large subunit of NoRC, binds to pRNAs. pRNA interacts with regulatory elements in the rDNA promoter, forms a DNA:RNA triplex, and is recognized by the DNA methyltransferase DNMT3b (Figure 1; Mayer et al., 2008; Bierhoff et al., 2010). Thus, the binding of NoRC to the rDNA promoter represses rDNA transcription through recruitment of histone modifying and DNA methylating enzymes (Santoro et al., 2002; Mayer et al., 2006; Guetg et al., 2010).

Interestingly, by using mature pRNAs to tether heterochromatin at nucleoli in embryonic stem cells, Savić et al. (2014) found that localized heterochromatin condensation of rDNA genes initiates establishment of highly condensed chromatin structures outside of the nucleolus. Meanwhile, the formation of such highly condensed, transcriptionally inactive heterochromatin promotes transcriptional activation of differentiation genes and loss of pluripotency of embryonic stem cells. NoRC safeguards genome stability by triggering heterochromatin formation at telomeres and centromeres (Postepska-Igielska et al., 2013). Whether and how pRNA and NoRC function together to maintain rDNA stability requires further investigation.

### LoNA Modulates rRNA to Promote Learning and Memory

**FIGURE 2:**
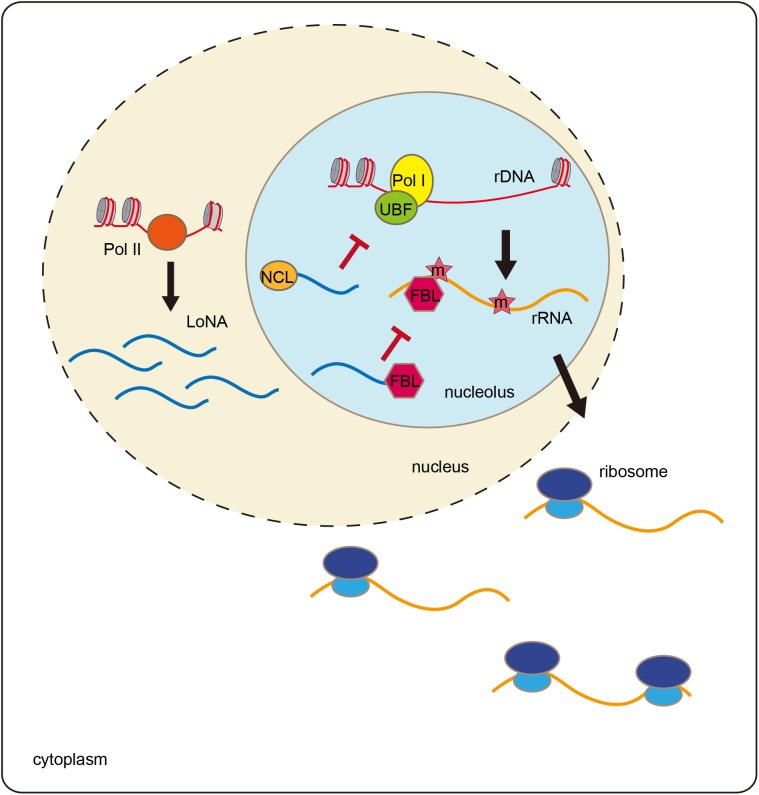
LoNA modulates rRNA and promotes learning and memory. LoNA is synthesized by Pol II. LoNA interacts with nucleolin (NCL) and inactivates the chromatin status of rDNA region via reducing the loading of UBF and Pol I to rDNA loci. Meanwhile, LoNA competes with snoRNAs to bind to FBL, thereby altering the methylation status of rRNAs. Therefore, by binding to both NCL and FBL proteins, LoNA suppresses rRNA production and alters ribosome heterogeneity.

When mice are trained with a Morris water maze, both rRNA and pre-rRNA levels are significantly elevated (Li et al., 2018). The trained mice exhibit decreased expression of the lncRNA LoNA in the hippocampus. LoNA is synthesized by Pol II and specifically enriched in nucleolus, and it can suppress the transcription of pre-rRNAs (Figure 2). The 5′ portion of LoNA interacts with nucleolin (NCL), while its 3′ portion contains a snoRNA that binds to fibrillarin (FBL). NCL can remodel rDNA loci, and can, therefore, modulate pre-rRNA transcription (Abdelmohsen and Gorospe, 2012; Durut and Saez-Vasquez, 2015). LoNA binds to NCL and inactivates the chromatin status of rDNA region. FBL is a component of C/D box small nucleolar ribonucleoproteins (snoRNPs), which direct 2′-*O*-methylation of rRNAs and participate in rRNA processing (Newton et al., 2003). LoNA competes with snoRNAs to bind to FBL, thereby altering the methylation status of rRNAs. By binding to both NCL and FBL proteins, LoNA suppresses rRNA production and alters ribosome heterogeneity (Li et al., 2018).

Nucleolar stress is accompanied by decreased rRNA synthesis and failures in ribosome biogenesis and functions, which are considered to be cellular stress events associated with aging and neurodegenerative diseases (Boulon et al., 2010; Wu et al., 2018). In patients with Alzheimer disease (AD), rRNA production decreases (Dönmez-Altuntaş et al., 2005). In an AD animal model, LoNA expression is significantly increased in the mouse brain, which is accompanied by decreased rRNA levels (Li et al., 2018). Mice lacking LoNA are more efficient in locating the hidden platform in Morris water maze tests than are the control animals. LoNA-deficient AD mice show rescue of the learning and memory defects compared to the control animals in Morris water mazes and in object context discrimination behavioral tests. These results suggest that hippocampal LoNA is involved in learning and memory and may represent a potential therapeutic target for AD treatment.

### SLERT Regulates DDX21 Rings Associated With Pol I Transcription

SLERT is a box H/ACA snoRNA-ended lncRNA (Xing et al., 2017). SLERT contains 694 nt and is highly expressed in many human cell lines, especially in human embryonic stem cells and ovarian carcinoma cells. SLERT mainly accumulates in the nucleolus, and its localization depends on its box H/ACA snoRNA ends. SLERT depletion results in decreased levels of the 18S and 28S rRNAs, indicating that SLERT promotes rRNA production.

Mass spectrometry (MS) data of SLERT-associated proteins identified DDX21, a DEAD-box RNA helicase that is involved in multiple steps of ribosome biogenesis (Holmström et al., 2008; Calo et al., 2015; Sloan et al., 2015). SLERT depletion enhances the interaction between DDX21 and Pol I by tightening the DDX21 rings surrounding Pol I complexes, thereby suppressing rDNA transcription (Xing et al., 2017). Dysregulated rRNA synthesis by Pol I is associated with uncontrolled cancer cell proliferation (Nguyen le et al., 2015). The interaction between SLERT and DDX21, therefore, represents a potential therapeutic target for future anti-cancer drug discovery (Peltonen et al., 2014).

### PAPAS Responds to Environment Stresses to Maintain rRNA Suppression

**FIGURE 3:**
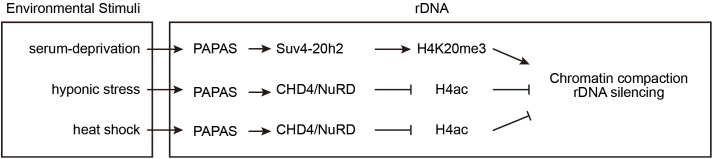
PAPAS responds to environmental stimuli to suppress rDNA transcription. At rDNA clusters, the lncRNA PAPAS directs Suv4-20h2 for repressive mark H4K20me3 deposition after growth factor deprivation. Upon hypotonic and heat-shock stresses, PAPAS recruits NuRD to rDNA and induces a nucleosomal “off” position to repress rDNA transcription.

Upon stress, cells utilize various strategies to suppress rDNA transcription to promote survival, for example, by inactivating certain transcription factors and inducing chromatin remodeling (Bierhoff et al., 2014; Holland et al., 2016). Furthermore, a class of lncRNAs, PAPAS, is expressed to inhibit pre-rRNA transcription (Figure 3). PAPAS is transcribed by RNA polymerase II from a fraction of the rDNA units in an antisense orientation, and, therefore, it is called “promoter and pre-rRNA antisense” (PAPAS) (Bierhoff et al., 2010, 2014). PAPAS comprises a heterogeneous population of 12–16 kb lncRNAs that are complementary to both the pre-rRNA coding region and the rDNA promoter. PAPAS responds to distinct stresses and modulates pre-rRNA synthesis accordingly.

In density-arrested or serum-deprived cells, pre-rRNA synthesis is suppressed and rDNA is enriched with H4K20me3 marks, while PAPAS is upregulated. After starved cells are refed with serum, the levels of H4K20me3 modifications and PAPAS decrease. RNA immunoprecipitation (RIP) experiments revealed that quiescence-induced PAPAS recruits Suv4-20h2 to transcription-competent rRNA genes to trigger H4K20me3 modification and chromatin compaction. Furthermore, siRNA-mediated knockdown of endogenous PAPAS decreased the level of H4K20me3 modifications, but not the level of H3K9me3 modifications (Bierhoff et al., 2014).

Hypo-osmotic stress also upregulates PAPAS and inhibits rDNA transcription. However, unlike serum deprivation, hypo-osmotic shock does not increase the Suv4-20h2 occupancy and H4K20me3 abundance at rDNA loci, but rather induces the degradation of Suv4-20h2 (Zhao et al., 2016a). The Mi-2/nucleosome remodeling and deacetylase factor (NuRD) is a multisubunit protein complex containing the HDAC1 histone deacetylase and the ATP-dependent remodeling enzyme CHD4 (Xue et al., 1998; Torchy et al., 2015). Upon hypo-osmotic stress, the elevated PAPAS associates with CHD4/NuRD and recruits them to rDNA regions where they deacetylate histone H4, remodel the promoter-bound nucleosomes, and reinforce transcriptional repression (Zhao et al., 2016a).

Similar to hypotonicity, heat shock also increases PAPAS expression, induces the degradation of Suv4-20h2, recruits NuRD to rDNA, and turns off transcription of pre-rRNA (Zhao et al., 2016b). Recent studies revealed the molecular mechanism of how PAPAS recruits CHD4/NuRD to rDNA. CHD4 is an RNA-binding protein that associates with both DNA and RNA via its N-terminal PHD and chromo-domains. Heat-shock elicits CHD4 dephosphorylation to facilitate its association with PAPAS (Zhao et al., 2018). PAPAS binds to the adjacent rDNA sequence via the formation of a DNA-RNA triplex, thereby directing CHD4/NuRD to rDNA, where it remodels the chromatin into a transcription refractory state (Zhao et al., 2016b, 2018).

### 5S-OT Plays a Cis Role in Regulating the Transcription of 5S rRNA and a Trans Role in Alternative Splicing of mRNAs

Unlike other Pol I-transcribed rRNAs, 5S rRNAs are transcribed by Pol III. 5S rRNA genes are clustered as tandem repeats with intergenic sequences, and they are located on distinct chromosomes (Ciganda and Williams, 2011). A number of studies have revealed Pol II binding sites adjacent to Pol III-transcribed genes, including the 5S rRNA genes (Oler et al., 2010; Hu et al., 2012). These cryptic Pol II transcripts may therefore modulate the transcription of neighboring 5S rRNAs.

Hu et al. (2016) identified a lncRNA, 5S rRNA overlapped transcripts (5S-OT), that is transcribed by RNA polymerase II and is complementary to the 5S rRNA. 5′ and 3′ rapid amplification of cDNA ends (RACE) experiments demonstrated that this transcript contains 847 and 354 nt in mice and humans, respectively. Chromatin immunoprecipitation (ChIP) experiments indicated that Pol II binds to the promoter at the 5S-OT transcription start site in both mouse and human cells. Inhibition of Pol II with α-amanitin results in a decreased level of 5S-OT transcripts, which further leads to a reduction of nascent 5S rRNAs. Consistently, knocking down 5S-OT by siRNAs also inhibits the production of nascent 5S rRNAs. It was suggested that in mammalian cells, the lncRNA 5S-OT associates with 5S rDNA clusters where it promotes the transcription of 5S rRNAs, thus providing a mechanism to couple Pol II and Pol III transcription.

Furthermore, human 5S-OT contains an antisense Alu element at its 3′ end (Hu et al., 2016). Alu is a primate-specific transposable element. The Alu element in the human 5S-OT gene belongs to the AluY subfamily (Batzer and Deininger, 2002). In human cells, 5S-OT regulates alternative splicing of multiple genes in trans via Alu/anti-Alu pairing with targeted genes and by interacting with the splicing factor U2AF65.

Therefore, the lncRNA 5S-OT modifies 5S rRNA and mRNAs via cis and trans mechanisms, respectively. Since 5S-OT is relatively conserved in eukaryotes from fission yeast to humans, it will be interesting to examine whether similar mechanisms are applicable in other organisms.

## Small Regulatory RNAs Inhibit Pre-rRNA Via The Nuclear RNAi Pathway

**FIGURE 4:**
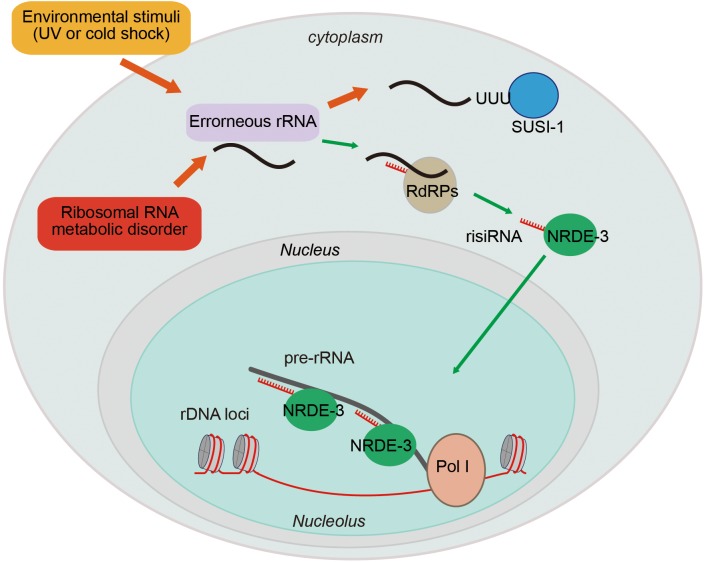
Ribosomal siRNAs silence pre-rRNAs via the nuclear RNAi pathway. rRNA biogenesis is a multistep process, including a series of pre-rRNA processing, folding, and modification steps. Endogenous metabolic stresses and environmental stimuli can cause RNA processing disorders, leading to the accumulation of erroneous RNAs. This accumulation leads to the recruitment of RdRPs to synthesize risiRNA, subsequently turning on the nuclear RNAi-mediated gene silencing pathway to inhibit pre-rRNA expression.

The gene silencing capacity of small interfering RNAs (siRNAs) was first described in *Caenorhabditis elegans* two decades ago (Fire et al., 1991). siRNAs silence complementary nucleic acids in both the cytoplasm and nucleus. Previous research has focused on the mechanism of siRNA-dependent regulation of mRNAs. In the cytoplasm, siRNAs can direct the degradation of targeted RNAs and inhibit protein translation (Ipsaro and Joshua-Tor, 2015). In the nucleus, siRNAs can guide heterochromatin formation and inhibit transcription elongation (Feng and Guang, 2013; Rechavi and Lev, 2017). Here, we will summarize our recent work that begins to illustrate the function of siRNAs in the regulation of ribosomal RNAs (Figure 4; Zhou et al., 2017a; Zhu et al., 2018).

Antisense ribosomal siRNAs (risiRNAs) are widely present in various organisms. In *Schizosaccharomyces pombe* lacking Cid14, rRNAs become substrates for the RNAi pathway, giving rise to siRNAs targeting rRNA (Buhler et al., 2008). In *Neurospora crassa*, DNA damage induces the expression of the Argonaute protein QDE-2 and a class of RNAs that interact with it (qiRNAs) from the ribosomal DNA locus (Lee et al., 2009).

In *C. elegans*, upon exposure to low temperature treatment or ultraviolet (UV) light, risiRNAs accumulate (Zhou et al., 2017b). risiRNAs are complementary to the 18S and 26S rRNAs, contain 22 nt, and start with a 5′ guanosine. risiRNAs belong to a class of 22G-RNAs that are synthesized by the RNA-dependent RNA polymerases (RdRPs) in *C. elegans*. risiRNAs associate with the Argonaute protein NRDE-3 and translocate to nucleolus, where they suppress pre-rRNA expression (Zhou et al., 2017b; Zhu et al., 2018).

Ribosomal siRNAs act to surveil erroneous rRNAs and maintain rRNA homeostasis. Misprocessed rRNAs are usually detected and degraded by multiple surveillance machineries, including the exosome and Trf4/Air2/Mtr4p polyadenylation (TRAMP) complexes (Schmidt and Butler, 2013). The exonuclease SUSI-1 (ceDIS3L2) is involved in the 3′–5′ degradation of oligouridylated rRNA fragments (Astuti et al., 2012; Zhou et al., 2017b). When the rRNA modification or processing steps are disrupted, or upon cold shock treatment or UV exposure, erroneous rRNAs are oligouridylated and recognized by RdRPs to generate risiRNAs via a poorly understood mechanism (Zhou et al., 2017b; Zhu et al., 2018). risiRNAs in turn silence rRNAs via the RNAi machinery to prohibit the accumulation of erroneous rRNAs.

Downregulation of rRNA transcription is one of the major strategies to preserve cellular homeostasis upon encountering stress conditions and to limit energy consumption under unfavorable conditions (Parlato and Liss, 2014). The risiRNA/RNAi-directed feedback loop, therefore, may compensate for dysfunctions in the exoribonuclease-engaged degradation of erroneous rRNAs. Consistently, when rRNA modification and processing steps are defective, the animals grow more slowly because of the presence of risiRNAs (Zhu et al., 2018).

## Perspectives

Ribosome biogenesis is tightly coordinated and controlled from the transcription of pre-rRNAs to the assembly of ribosomes, a process that is influenced by many developmental programs and environmental stress challenges (McStay, 2016; Sloan et al., 2017). Cells respond to these signals by modulating the transcription, processing, maturation of rRNAs and the assembly and usage of ribosomes. Defects or disorders in any of these steps lead to a number of human diseases. In addition to protein factors, small and long regulatory RNAs also play important roles in the regulation of pre-rRNA transcription and rRNA maturation. The regulatory RNAs may act to sense developmental signals and environmental stresses. For example, PAPAS can sense nutrition deprivation, heat-shock, and hypo-osmotic stresses. risiRNAs respond to cold shock and UV and further down regulate pre-rRNA transcription. risiRNAs also surveil the fidelity and precision of rRNA modifications and processing to avoid the accumulation of erroneous rRNAs expressed during the development of organisms. More interestingly, the lncRNA LoNA is involved in learning and memory by modulating rRNA transcription.

Many questions still remain to be addressed to fully understand the mechanisms and roles of non-coding RNAs in anti-stress pathways and rRNA regulation. For example, how do these lncRNAs surveil distinct environmental stresses? Cold shock could induce untemplated addition of oligouridylation at the 3′ ends of 26S rRNAs to elicit the production of risiRNAs (Zhou et al., 2017b). Heat-shock elicits CHD4 dephosphorylation to facilitate its association with PAPAS (Zhao et al., 2018). Were these surveillance mechanisms conserved among different organisms during evolution? Do poikilotherms and homeotherms use similar mechanisms to sense temperature alterations in the environment? Beside nuclear non-coding RNAs, cytoplasmic lncRNAs are frequently bound to and degraded at ribosomes (Carlevaro-Fita et al., 2016). Whether these cytoplasmic lncRNAs can in turn regulate rRNAs need further investigation. In particular, what are the biological roles of non-coding RNAs in regulating rRNAs during developmental processes? With new emerging technologies, many novel discoveries will help to answer these important questions.

## Author Contributions

All authors listed have made a substantial, direct and intellectual contribution to the work, and approved it for publication.

## Conflict of Interest Statement

The authors declare that the research was conducted in the absence of any commercial or financial relationships that could be construed as a potential conflict of interest.
